# The Secretions as Guides to Treatment

**Published:** 1872-04

**Authors:** H. W. Fuller

**Affiliations:** Physician to St. George’s Hospital, London


### THE SECRETIONS AS GUIDES TO TREATMENT.

BY DR. H. W. FULLER, PHYSICIAN TO ST. GEORGE'S HOSPITAL, LONDON.

[Failure of treatment frequently depends upon a neglect to examine carefully, the character of the secretions. Our diagnosis of the precise nature of the disorder, may be perfect, but this alone does not always suffice for success in its treatment.]

Let me take, as an example, the case of a man at present in the Cambridge ward, who was admitted suffering from anasarca, occasioned by a weak and dilated heart. In addition to extensive dropsy of the extremities, he had urgent dyspnœa, congestion of the lungs, effusion into the pleural cavity, engorgement of the liver, with pale-coloured fæcal evacuations, and scanty, loaded, non-albuminous urine, tympanitic distension of the abdomen, and slight effusion into the abdominal cavity—the ordinary assemblage of symptoms which result from long-continued interference with the central organ of the circulation. Before I saw him he had taken salines with digitalis, squills, scoparium, and other diuretics; but his urine had not increased in quantity, and his symptoms had been gradually increasing in severity. This was his condition when he was admitted into the hospital. Having regard to the pale color of his motions, I felt convinced that no real good could be effected unless a free flow of bile could be induced; and that the failure of the treatment he had undergone before admission into the hospital depended principally, if not solely, upon the neglect to stimulate the action of the liver and the secreting apparatus of the bowels. I therefore, prescribed five grains of the compound digitalis pill—containing three grains of blue pill, a grain of squills, and half a grain of digitalis—three times a day; and within three days he had begun to improve. Before the end of a week the motions had assumed a healthy color, the quantity of urine was trebled, the flatulence had almost disappeared, the anasarca was rapidly decreasing, and the dyspnœa subsiding. Indeed, it was obvious that the very medicines which before had proved inoperative, had become active agents for good as soon as the secreting apparatus of the liver and bowels had been stimulated to healthy action.

Take another class of cases. It sometimes happens that patients are admitted suffering from ague, with which they have been afflicted for many weeks. They have taken quinine perseveringly, but have not obtained relief. On examination the cause is at once apparent. Their internal organs are in a state of engorgement, and consequently are sluggish; their bowels are costive, and their motions unhealthy. In these cases the administration of remedies calculated to stimulate the viscera to action is all that is needed to restore them to health. Two or three doses of colocynth and rhubarb, in combination with quinine, will at once arrest the disease, which weeks of mere quinine taking had failed even to control.

In many forms of dyspepsia, the same holds good. The secretions of the stomach and bowels are disordered, the liver is gorged, and the tongue is covered with a yellow fur. The patient has been purged by means of senna, colocynth, or rhubarb; alkalies and alkaline earths, together with vegetable bitters, have been given; possibly the mineral acids have also been tried; moderately strict dieting has been had recourse to; and other expedients have been adopted, but in vain. The disagreeable taste in the mouth, the acidity, waterbrash, flatulence, drowsiness after meals, and restlessness at night, continue unabated. And what is the cause of this failure of treatment? Though the bowels act regularly, the motions are pale and lumpy, or else dark-colored and offensive; and the urine is scanty and high-colored, and loaded with lithates. Cases such as these often come before us in the wards, and occur still more frequently in private consulting practice. They convey a lesson which must not be forgotten, even though healthy. Dogs, with artificially made billiary fistulæ, do not appear to secrete an increased quantity of the bile under the influence of moderate doses of calomel. The lesson which they teach, and which you will do well to remember, is, that so long as the secretory apparatus is inactive or out of order, the remedies which are ordinarily most efficacious in relieving the symptoms of dyspepsia, are of little avail; whereas they exert their beneficial influence directly that disorder is rectified, and secretion is re-established. In cases such as these, a few doses of calomel, combined with opium, if necessary, will, in a few days, effect a change for the better, which cannot be brought about by other means in as many weeks. Under their influence the motions will lose their offensive odor, and assume a healthy color, the tongue will clean, the urine will become clear, the symptoms of acidity and discomfort will pass off, and your patient will give you credit for affording him relief. When this healthy condition of the secretions has been attained, you need have little anxiety as to your patient's recovery, for the remedies which had previously proved ineffective, will speedily quiet the irritability of the stomach, increase its tone, and restore your patient to health.

Let me take yet one more class of cases—the most common, perhaps, with which we have to do, and that in which, perhaps more than in any other, a want of proper regard to the secretions leads inevitably to unsuccessful practice. I refer to cases of so-called debility, in which stimulants, high feeding, and tonics are constantly recommended, and are often fruitlessly had recourse to. The cases to which these observations apply are of every imaginable description. In private practice it often happens that a person, not otherwise out of health, is accidentally deprived of his usual exercise; and his appetite being unimpaired, he takes more food than is absolutely required for the repair of the body. The wear and tear of his tissues being much less than usual, owing to his inactivity, and the supply of fresh materials, owing to his unimpaired appetite, being in excess of the actual requirements of the body, one of two things must necessarily happen: either his excretory organs must do an unusual amount of work, and throw out of the system the whole of the matters which have been taken in excess of the actual requirements of the tissues, or the surplus materials must accumulate in the blood, alter its quality, and oppress the nervous system. The alternative is not doubtful. When the blood is surcharged with materials which, however good and nutritious, are yet in excess of the requirements of nutrition, the nervous centres are oppressed; and not only does langor, or general debility, as it is termed, occur, but the liver, kidneys, and other secretory organs become gorged and sluggish, the urine becomes scanty, and the motions become clay-colored, or else dark and offensive. In private practice, cases such as these are constantly met with, in which, notwithstanding the unhealthy state of the secretions, tonics have been given for months, together with stimulants and every variety of strong food, and in which a few days of active purgation, some alterative doses of mercury, enforced exercise, and a restricted diet, by leading to the elimination of the surplus materials, and so to a purification of the blood, do more than all the previous tonics and rich feeding to put an end to the patient's langor, and restore his physical and mental power. In hospital practice, these particular cases less frequently come before us; but in another form, and under other circumstances, similar instances not unfrequently present themselves. In the Roseberry Ward, at the present time, is a stout, strong-looking, hysterical servant girl, Sarah Fleetwood, suffering from amenorrhœa. She had undergone treatment for a considerable period before admission, and throughout had taken iron in various forms, but had not obtained relief. On admission, she complained of extreme langor and debility, and of utter loss of appetite. Her tongue was clean, and her bowels were said to be regular; but her urine was scanty and loaded with lithates; on examination, her motions proved to be very scanty, and almost white, lumpy, and offensive, and had been so, according to her account, throughout her illness. She is a perfect type of the class of cases which I have been endeavoring to describe. She is oppressed by the presence in the blood of matters which ought long since to have been eliminated, but which the inactivity of her secretory organs has caused to be retained in the system; weak, in the sense in which a healthy person on the eve of a bilious attack is weak, but in no other; weak from a cause which no strengthening food and no tonics will remove, nay, rather which they will tend to aggravate, and which protracted semi-starvation or the skilled aid of the physician is required to rectify. The iron and other tonics, the port wine and other stimulants, which the poor girl had taken prior to her admission into the hospital, only tended still further to surcharge her system with materials which it was incapable of assimilating—to render her blood more noxious to the brain and nervous centres, and thus to increase her langor and aggravate her suffering. Yet these very remedies have nearly effected her cure, now that healthy secretion has been re-established. Already, under the influence of a few doses of calomel and colocynth, the urine, instead of being scanty and loaded, has become abundant, and in every respect healthy; the motions are no longer pale and lumpy, but have become normal in character; color has returned to her lips; she sleeps more quietly, feels stronger and less languid; and no longer exhibits a repugnance to food. And now that the iron is able to do its work, its beneficial influence will soon be manifested still more decidedly, and the menstrual discharge will speedily return. In a precisely similar case which I saw with Sir Charles Locock, many years ago, the symptoms did not yield until after a protracted course of calomel; and experience justifies me in warning you that in several forms of amenorrhœa, and in many of the so-called cases of general debility, a deranged condition of the alvine secretions lie at the very root of the symptoms, and that all remedies will fail to relieve the patient until steps have been taken to rectify them. Purgation may or may not be necessary, and the aid of calomel may or may not be needed; but if either or both prove requisite to attain the desired object, they must be used without hesitation. No theoretical considerations must be permitted to countervail such clear and unmistakable indications for treatment. Experience confirms what theoretical considerations would lead us to expect, namely, that the true mode of restoring strength, under the conditions we are now considering, is, not by administering food and tonics, which the patient is incapable of assimilating and making use of; nor is it by abstaining from administering alterative or aperient remedies, lest by so doing we should weaken the patient—both of which courses can only tend to continued mal-nutrition and a gradually-increasing failure of strength—but rather to endeavor, by appropriate means, and at whatever risk of present discomfort to the patient, to re-establish healthy secretion; and thus place him in a position to profit by wholesome food, the natural restorative of health and strength.—*St. George's Hospital Reports, vol.* 5, *p.* 17. —*Braithwaite's Retrospect, January*, 1872.

